# Components of 24-hour movement behavior and self-reported physical fitness in Ibero-American preschoolers, children, and adolescents during the COVID-19 pandemic

**DOI:** 10.3389/fpubh.2026.1837281

**Published:** 2026-06-11

**Authors:** Letícia de Borba Schneiders, José Francisco López-Gil, Sofia Fernandez-Gimenez, Enrique Pintos-Toledo, Anelise Reis Gaya, Javier Brazo-Sayavera

**Affiliations:** 1 Graduate Program in Epidemiology, Federal University of Pelotas, Pelotas, Brazil; 2 Graduate Doctorate Program in Physical Activity and Sports Sciences, Universidad Pablo de Olavide, Seville, Spain; 3 School of Medicine, Universidad Espíritu Santo, Samborondón, Ecuador; 4 Faculty of Health Sciences, Universidad Autónoma de Chile, Temuco, Chile; 5 Higher Institute of Physical Education, University of the Republic, Rivera, Uruguay; 6 Northeast Regional University Center, PDU EFISAL, University of the Republic, Rivera, Uruguay; 7 School of Physical Education and Physiotherapy, Federal University of Pelotas, Pelotas, Brazil; 8 Graduate Program in Human Movement Sciences, Federal University of Rio Grande do Sul, Porto Alegre, Brazil; 9 Department of Sports and Computer Science, Universidad Pablo de Olavide, Seville, Spain

**Keywords:** physical condition, physical activity, sedentary behavior, sleep, youth

## Abstract

**Objective:**

To evaluate the association between the component of 24-h movement behaviors (24HMB) (physical activity—PA; screen time—ST; sleep) and self-reported physical fitness in Ibero-American preschoolers, children, and adolescents during the Coronavirus Disease 2019 (COVID-19) pandemic.

**Methods:**

This cross-sectional study included 1,077 participants (3–17 years old) from Spain, Brazil, and Uruguay. The components of 24HMB were classified according to the Canadian Guidelines and the World Health Organization. Physical fitness was assessed using the International Physical Fitness Scale and dichotomized into high (good/very good) versus low (very poor/poor/medium) for general fitness, cardiorespiratory fitness, muscular fitness, speed/agility, and flexibility. Associations were assessed using generalized linear mixed models, reporting odds ratios (OR) and 95% confidence intervals (CI), adjusted for sex, age, and breadwinners’ educational level, with country as a random effect. Analyses were conducted in SPSS 25.0.

**Results:**

In preschoolers, not meeting PA recommendations was associated with low general physical fitness (OR = 3.44; 95% CI: 1.20–9.88). In children, not meeting PA recommendations was associated with low general physical fitness (OR = 2.13; 95% CI: 1.29–3.53), muscular fitness (OR = 1.87; 95% CI: 1.12–3.12), and speed/agility (OR = 2.09; 95% CI: 1.25–3.51). Not meeting sleep recommendations was associated with muscular fitness only in partially adjusted models (OR = 1.91; 95% CI: 1.11–3.18). In adolescents, not meeting PA recommendations was associated with low general physical fitness (OR = 3.53; 95% CI: 1.69–7.41), cardiorespiratory fitness (OR = 3.52; 95% CI: 1.41–7.26), speed/agility (OR = 3.36; 95% CI: 1.54–7.32), and flexibility (OR = 2.94; 95% CI: 1.53–5.64). Not meeting ST recommendations was associated with lower odds of high cardiorespiratory fitness (OR = 0.35; 95% CI: 0.12–0.96). In age-stratified analyses, meeting fewer movement behavior recommendations was associated with poorer physical fitness among children and adolescents, although pooled analyses showed no significant associations.

**Conclusion:**

Physical activity was the component of the 24HMB model most consistently associated with better self-reported physical fitness among Ibero-American youth, with stronger associations in older age groups. Sleep and ST showed less consistent associations, and low adherence to the recommendations was associated with poorer outcomes. These findings reinforce the importance of integrated 24-h strategies with a focus on physical activity.

## 1 Introduction

The Coronavirus Disease 2019 (COVID-19) pandemic had devastating consequences for the health of the entire population, while also negatively affecting education and the economy in many countries (1, 2). Remote learning was implemented in schools for several months, compromising children’s and adolescents’ participation in school and extracurricular activities, such as physical education classes and sports programs (3). The restrictions imposed during the pandemic had negative impacts on the behavior of school-aged children and adolescents, including reduced levels of physical activity (PA), increased screen time (ST), and disruptions in sleep patterns (4, 5). Restriction measures and school closures varied between countries, affecting children’s health and development differently. In Latin America, the Pan American Health Organization reported an educational crisis, and the United Nations Children’s Fund identified increased stress and anxiety among adolescents during the pandemic. These changes raised important concerns about the general health and well-being of children and adolescents during this period (6, 7).

PA, sedentary behavior, and sleep are important and interdependent health behaviors (8). Therefore, they should be examined in an integrated manner to promote greater health benefits. The 24-h movement behavior guidelines (24HMB) include recommendations for three daily behaviors: PA, recreational ST, and sleep (9–11). Although each behavior provides benefits when considered independently, the balanced combination of these behaviors throughout the day represents an optimal condition for health (12, 13). Children and adolescents who meet the 24HMB recommendations tend to have healthier cardiometabolic and adiposity indicators compared to those who do not meet these guidelines (52). According to the review by Zhang et al. (14), the number of studies assessing 24HMB during the COVID-19 pandemic remains limited compared to studies examining these behaviors in isolation. In this regard, new evidence is essential for researchers, practitioners and policymakers to develop strategies that encourage behavioral change and reduce the health risks intensified during the pandemic.

Physical fitness is an important indicator of health (15). Children and adolescents with adequate levels of physical fitness have a lower risk of developing diseases such as type 2 diabetes mellitus and hypertension. Furthermore, good levels of physical fitness during childhood and adolescence are associated with the adoption of more active and healthier lifestyles in adulthood (16, 17). The interaction between adequate levels of PA, reduced exposure to sedentary behavior, and sufficient sleep may positively influence physiological adaptations related to cardiorespiratory fitness, muscular strength, and body composition (18, 19). Thus, promoting PA, reducing excessive ST, and ensuring adequate sleep are important strategies for the development of physical fitness (20). There is already evidence that shows positive associations between each healthy daily behavior and higher levels of physical fitness among children and adolescents (21–23).

However, evidence on the association between 24HMB and physical fitness during the COVID-19 pandemic remains limited, particularly in Ibero-American countries, where sociocultural and policy differences can influence these behaviors (24–26). Therefore, this study evaluated the association between the 24HMB components (PA, ST and sleep) and physical fitness among Ibero-American preschoolers, children, and adolescents during the COVID-19 pandemic.

## 2 Methods

### 2.1 Study design and participants

The present research evaluated a cross-sectional sample of Brazilian, Uruguayan, and Spanish children aged 3 to 17 years. Parents or guardians were recruited through social media such as social networks. A snowball sampling strategy was adopted to distribute the online survey in each country. Participants were asked to provide informed consent, and the objectives of the study were explained to them. The online survey took approximately 15 min to complete. The data were collected over 15 days in the three countries (in March in Spain, in April in Brazil and in November in Uruguay, all in 2020). Preschoolers, children, and adolescents aged 3–17 years were selected for this study. Of the 1,186 participants assessed, 99 were removed because they were <3 years or >17 years. An additional 10 participants were removed due to missing values. In total, 1,077 participants were included in the final analysis. This research was approved by the Ethics Committee of the Catholic University of Murcia in Spain (Registration: CE112001), the Technical University of Paraná in Brazil (Registration: 4.275.232) and the Universidad de la República in Uruguay (Registration: 311170–000673-19). Parents or guardians invited to respond to the online survey should be isolated during the week before their response, so information can be provided about restrictions on the spread of COVID-19. Various information, such as the nationality, age, and sex of the children, was obtained from the parents.

### 2.2 Components of 24-h movement behaviors

#### 2.2.1 Physical activity

PA was measured using the following question: “Normally, how many days was your child physically active, for a total of at least 60 min?” The responses varied between 0 and 7 days per week, with increments of 1 day. The classification of PA was defined as “follows recommendations” for those who responded to do 60 min of PA per day, 7 days a week, and “does not follow recommendations” for those who did not comply with this requirement (10, 11).

#### 2.2.2 Screen time

Respondents were also asked to report the amount of time their children spent performing various sedentary activities using screens. The following questions were applied during the week and on weekends: [1] “How many hours a day, in free time, does your child usually spend watching TV, videos (including YouTube or similar services), DVDs?” [2] “How many hours a day, in free time, does your child usually spend playing games on a computer, game console, tablet, smartphone or other electronic device (not including movement or fitness games)?” and [3] “How many hours per day of your child’s free time does he or she typically spend using electronic devices such as computers, tablets, or smartphones for other purposes (e.g., homework, sending emails, tweeting, facebooking, chatting, surfing the internet)?” A weighted sum of the three questions was performed, considering 5 days of the week and 2 days of the weekend. ST was categorized according to the Canadian Guidelines on Screen Time in Children and Youth (11) as “does not meet guidelines” for children and adolescents with time >2 h/d and “meets guidelines” for children with time ≤2 h/d. And according to the recommendations of the World Health Organization (WHO) guidelines for ST in children under 5 years (10), as “does not meet guidelines” for preschoolers with time >1 h/d and “meets guidelines” for preschoolers with time ≤1 h/d.

#### 2.2.3 Duration of sleep

To assess sleep, respondents were asked the following questions on weekdays and weekends: “What time does your child usually go to bed?” and “What time does your child usually wake up?” A calculation was performed to determine the average daily sleep duration for each participant as follows: [(average nighttime sleep duration on weekdays × 5) + (average nighttime sleep duration on weekends × 2)]/7. Sleep was classified as “meets sleep guidelines” for responses from preschoolers aged 3 to 4 years with a sleep range of 10 to 13 h, for children aged 5 to 13 years with a sleep range of 9 to 11 h, and for adolescents aged 14 to 17 years with a sleep range of 8 to 10 h. Participants who responded outside these ranges were classified as “does not meet sleep guidelines” according to the recommendations for 24HMB for sleep (27) and the WHO guidelines for the early years (10).

### 2.3 Self-reported physical fitness

The International Fitness Scale (IFIS) was used to assess self-reported physical fitness. The IFIS consists of five Likert scale questions that ask about perceived general fitness, cardiorespiratory fitness, muscular fitness, speed/agility and flexibility compared to your friends (28). The Likert scale is composed of five categories (1-very poor; 2-poor; 3-medium; 4-good and 5-very good). In the present study, the results were recategorized into two categories (1, 2 and 3—not high; 4 and 5—high). This categorization was performed to increase the statistical power.

### 2.4 Statistical analysis

To describe the data, frequency (percentage) was used for categorical variables. Participants were divided into three groups: Spanish, Brazilian, and Uruguayan. To identify possible associations between 24HMB components and indicators of physical fitness, a generalized linear mixed model (GLMM) was used, presenting the results as odds ratios (OR) and 95% confidence intervals, adjusted for sex, age, and breadwinners’ educational level. To control for data dependency, a second model was tested in which countries were included as a random-effect variable to account for potential unexplained variation among the three countries. The same analytical approach was applied to examine the associations between adherence to none, one, two, or three components of 24HMB and self-reported physical fitness indicators. As a complementary analysis, age-stratified analyses were performed. Due to the low prevalence of participants meeting all three behavioral recommendations, adherence to two components was used as the reference category in the stratified models. To quantify between-country variability, the intraclass correlation coefficient (ICC) and the median odds ratio (MOR) were calculated based on the variance of the country-level random intercept obtained from the multilevel logistic models. The ICC was estimated using the latent variable approach, assuming a level-1 residual variance fixed at 3.29, according to the formula: ICC = σ2/(σ2 + 3.29), where σ2 represents the between-country variance. The MOR was calculated to express contextual heterogeneity on the OR scale using the equation: MOR = exp. (0.6745 × √2 × σ2), representing the median increase in the odds of the outcome when comparing two individuals with identical characteristics from two randomly selected countries. The Statistical Package for Social Sciences v.25 (SPSS, IBM Corp., Armonk, NY, United States) was used to perform all analyses. A *p*-value <0.05 was considered statistically significant.

## 3 Results

Table 1 presents the descriptive characteristics of the participants evaluated by country. Regarding the age range, preschoolers are more represented in Spain (14.1%) and Brazil (15.1%), children predominate in Uruguay (89.0%), while adolescents are more common in Spain (46.9%). Spain has a higher proportion of participants with low educational attainment of the family provider (40.3%), while Brazil has a majority with high educational attainment (72.8%). Regarding behaviors, the majority of participants do not meet the ST recommendations (94.6%), with 100% of Uruguayans in this group. Uruguay has the highest proportion of participants who meet the PA recommendations (65.8%), while Brazil has the lowest (22.5%). Regarding sleep, the lowest proportion of participants who meet the recommendations is in Uruguay (50.7%), while Brazil has the highest (76.4%).

**Table 1:** Descriptive characteristics.

Variable	Total	Spain	Brazil	Uruguay
	*n* = 1,077	*n* = 533	*n* = 471	*n* = 73
Age				
Preschoolers	148 (13.7)	75 (14.1)	71 (15.1)	2 (2.7)
Children	509 (47.3)	208 (39.0)	236 (50.1)	65 (89.0)
Adolescents	420 (39.0)	250 (46.9)	164 (34.8)	6 (8.2)
Sex				
Male	576 (53.5)	272 (51.0)	268 (56.9)	36 (49.3)
Female	501 (46.5)	261 (49.0)	203 (43.1)	37 (50.7)
Breadwinner’s educational level				
Low	301 (27.9)	215 (40.3)	68 (14.4)	18 (24.7)
Medium	216 (20.1)	141 (26.5)	60 (12.7)	15 (20.5)
High	560 (52.0)	177 (33.2)	343 (72.8)	40 (54.8)
Screen time				
No meeting	1,019 (94.6)	494 (92.7)	452 (96.0)	73 (100.0)
Meeting	58 (5.4)	39 (7.3)	19 (4.0)	0 (0.0)
Physical activity				
No meeting	772 (71.7)	382 (71.7)	365 (77.5)	25 (34.2)
Meeting	305 (28.3)	151 (28.3)	106 (22.5)	48 (65.8)
Sleep				
No meeting	325 (30.2)	178 (33.4)	111 (23.6)	36 (49.3)
Meeting	752 (69.8)	355 (66.6)	360 (76.4)	37 (50.7)
General physical fitness				
Not high	359 (33.3)	159 (29.8)	166 (35.2)	34 (46.6)
High	718 (66.7)	374 (70.2)	305 (64.8)	39 (53.4)
Muscular fitness				
Not high	380 (35.3)	194 (36.4)	138 (29.3)	48 (65.8)
High	697 (64.7)	339 (63.6)	333 (70.7)	25 (34.2)
Cardiorespiratory fitness				
Not high	370 (34.4)	194 (36.4)	131 (27.8)	45 (61.6)
High	707 (65.6)	339 (63.6)	340 (72.2)	28 (38.4)
Speed/agility				
Not high	349 (32.4)	195 (36.6)	118 (25.1)	36 (49.3)
High	728 (67.6)	338 (63.4)	353 (74.9)	37 (50.7)
Flexibility				
Not high	431 (40.0)	231 (43.3)	154 (32.7)	46 (63.0)
High	646 (60.0)	302 (56.7)	317 (67.3)	27 (37.0)
24HMB				
None	218 (20.2)	117 (22.0)	90 (19.1)	11 (15.1)
One	618 (57.4)	297 (55.7)	282 (59.9)	39 (53.4)
Two	226 (21.0)	109 (20.5)	94 (20.0)	23 (31.5)
All three	15 (1.4)	10 (1.9)	5 (1.1)	0 (0.0)

24HMB, 24-h movement behaviors. Values are expressed as the absolute (relative) frequency.

Table 2 presents the associations between the 24HMB components and low self-reported physical fitness in preschoolers. In Model 1 (adjusted for sex, age, and level of education of the family provider), not meeting the PA recommendations was associated with higher odds of low general physical fitness (OR = 3.61; 95% CI: 1.16–11.22). After further adjustment for countries (Model 2), the association between not meeting PA recommendations and low general physical fitness remained significant (OR = 3.44; 95% CI: 1.20–9.88). ST and sleep were not significantly associated with any component of physical fitness. Although the random effect variance was not statistically significant, ICC estimates suggested that a non-negligible proportion of the variability (10.1 to 16.5%) was attributable to country-level differences in the models for PA.

**Table 2:** Associations between 24HMB and self-reported physical fitness in preschoolers.

Predictor	Outcome	Model 1 OR (95% CI)	Model 2 OR (95% CI)	Random effects (country)		
Preschoolers				Variance (SE)	MOR	ICC (%)
Behavior (Ref: meeting)						
Screen time	General	0.83 (0.15–4.67)	0.54 (0.11–2.54)	0.54 (0.82)	2.02	14.0
	Muscular fitness	0.45 (0.13–1.63)	0.47 (0.09–2.52)	0.42 (0.58)	1.86	11.3
	Cardiorespiratory fitness	0.55 (0.15–2.00)	0.60 (0.18–1.95)	0.28 (0.63)	1.66	7.8
	Speed/agility	0.70 (0.18–2.71)	0.94 (0.18–4.84)	0.21 (0.47)	1.55	6.0
	Flexibility	1.45 (0.25–8.40)	1.62 (0.28–9.48)	0.09 (0.53)	1.33	2.7
Physical activity	General	3.61 (1.16–11.22)*	3.44 (1.20–9.88)*	0.38 (0.68)	1.80	10.4
	Muscular fitness	1.75 (0.82–3.76)	1.81 (0.62–5.29)	0.65 (0.62)	2.16	16.5
	Cardiorespiratory fitness	2.35 (0.89–6.25)	2.34 (0.78–7.04)	0.37 (0.70)	1.79	10.1
	Speed/agility	1.36 (0.77–3.53)	1.74 (0.61–4.97)	0.44 (0.78)	1.88	11.8
	Flexibility	1.09 (0.37–3.21)	1.12 (0.34–3.68)	0.57 (0.74)	2.06	14.8
Sleep	General	0.73 (0.21–2.57)	0.89 (0.31–2.56)	0.46 (0.77)	1.91	12.3
	Muscular fitness	0.51 (0.20–1.27)	0.43 (0.12–1.52)	0.06 (0.38)	1.26	1.8
	Cardiorespiratory fitness	0.69 (0.20–2.48)	0.73 (0.25–2.10)	0.82 (1.05)	2.37	20.0
	Speed/agility	0.86 (0.36–2.06)	0.79 (0.24–2.60)	0.09 (0.51)	1.33	2.7
	Flexibility	0.87 (0.26–2.92)	0.88 (0.28–2.78)	0.16 (0.48)	1.47	4.6

OR, odds ratio; CI, confidence interval; SE, standard error; MOR, median odds ratio; ICC, intraclass correlation coefficient; ≈0, country-level variance was estimated as redundant indicating no between-country variability. Model 1, Adjusted for sex, age, breadwinner’s educational level; Model 2, Adjusted for Model 1 + Countries. Significant values for *p* < 0.05.

*p < 0.05. **p < 0.001.

Table 3 presents the associations between 24HMB components and self-reported physical fitness in children. In Model 1, failure to meet PA recommendations was associated with a higher likelihood of low general physical fitness (OR = 1.59; 95% CI: 1.05–2.42), while failure to meet sleep recommendations was associated with low muscle fitness (OR = 1.91; 95% CI: 1.11–3.18).

**Table 3:** Associations between 24HMB and self-reported physical fitness in children.

Predictor	Outcome	Model 1 OR (95% CI)	Model 2 OR (95% CI)	*Random effects (country)*		
Children				Variance (SE)	MOR	ICC (%)
Behavior (Ref: meeting)						
Screen time	General	0.71 (0.26–1.95)	0.61 (0.20–1.84)	0.31 (0.39)	1.70	8.6
	Muscular fitness	1.13 (0.37–3.49)	0.91 (0.28–2.94)	0.59 (0.55)	2.08	15.2
	Cardiorespiratory fitness	1.49 (0.47–4.66)	1.25 (0.35–4.47)	0.85 (1.01)	2.41	20.5
	Speed/agility	2.12 (0.54–8.24)	1.86 (0.47–7.30)	0.03 (0.09)	1.18	0.9
	Flexibility	1.54 (0.50–7.79)	1.32 (0.40–4.05)	0.36 (0.36)	1.77	9.9
Physical activity	General	1.59 (1.05–2.42)*	2.13 (1.29–3.53)*	0.01 (0.07)	1.10	0.3
	Muscular fitness	1.15 (0.74–1.80)	1.87 (1.12–3.12)*	1.07 (0.85)	2.68	24.5
	Cardiorespiratory fitness	1.05 (0.69–1.59)	1.59 (0.97–2.60)	0.06 (0.30)	1.26	1.8
	Speed/agility	1.42 (0.92–2.19)	2.09 (1.25–3.51)*	0.27 (0.27)	1.64	7.6
	Flexibility	0.90 (0.62–1.36)	1.22 (0.78–1.89)	0.02 (0.14)	1.14	0.6
Sleep	General	1.10 (0.65–1.88)	0.89 (0.50–1.57)	0.11 (0.20)	1.37	3.2
	Muscular fitness	1.91 (1.11–3.18)*	1.31 (0.72–2.37)	0.58 (0.49)	2.07	15.0
	Cardiorespiratory fitness	1.66 (0.98–2.80)	1.13 (0.63–2.04)	0.46 (0.42)	1.91	12.3
	Speed/agility	1.29 (0.75–2.23)	0.92 (0.52–1.62)	0.31 (0.32)	1.70	8.6
	Flexibility	1.60 (0.98–2.61)	1.16 (0.68–1.97)	0.42 (0.41)	1.86	11.3

OR, odds ratio; CI, confidence interval; SE, standard error; MOR, median odds ratio; ICC, intraclass correlation coefficient; ≈0, country-level variance was estimated as redundant indicating no between-country variability. Model 1, Adjusted for sex, age, breadwinner’s educational level; Model 2, Adjusted for Model 1 + Countries. Significant values for *p* < 0.05.

*p < 0.05. **p < 0.001.

After adjusting for country (Model 2), failure to meet PA recommendations was significantly associated with low general physical fitness (OR = 2.13; 95% CI: 1.29–3.53), muscle fitness (OR = 1.87; 95% CI: 1.12–3.12), and speed/agility (OR = 2.09; 95% CI: 1.25–3.51). ICC estimates indicated substantial variability between countries for muscle fitness (24.5%), demonstrating that the association with PA may be influenced by national context.

Table 4 shows the associations between the 24HMB components and low self-reported physical fitness in adolescents. In Model 1, not meeting PA recommendations was associated with higher odds of low general physical fitness (OR = 3.63; 95% CI: 1.75–7.52), cardiorespiratory fitness (OR = 3.15; 95% CI: 1.47–6.72), speed/agility (OR = 3.23; 95% CI: 1.46–7.13), and flexibility (OR = 2.64; 95% CI: 1.48–4.72).

**Table 4:** Associations between 24HMB and self-reported physical fitness in adolescents.

Predictor	Outcome	Model 1 OR (95% CI)	Model 2 OR (95% CI)	*Random effects (country)*		
Adolescents				Variance (SE)	MOR	ICC (%)
Behavior (Ref: meeting)						
Screen time	General	0.64 (0.26–1.58)	0.62 (0.25–1.54)	0.03 (0.06)	1.18	0.9
	Muscular fitness	1.55 (0.58–4.11)	1.56 (0.58–4.19)	0.17 (0.56)	1.48	4.9
	Cardiorespiratory fitness	0.35 (0.12–1.02)	0.35 (0.12–0.96)*	0.05 (0.60)	1.24	1.5
	Speed/agility	0.69 (0.26–1.86)	0.73 (0.27–1.98)	0.03 (0.19)	1.18	0.9
	Flexibility	0.97 (0.37–2.66)	1.02 (0.37–2.80)	0.09 (1.55)	1.33	2.7
Physical activity	General	3.63 (1.75–7.52)*	3.53 (1.69–7.41)**	0.02 (0.05)	1.14	0.6
	Muscular fitness	1.72 (0.92–3.18)	1.79 (0.95–3.36)	0.16 (0.34)	1.47	4.6
	Cardiorespiratory fitness	3.15 (1.47–6.72)*	3.52 (1.41–7.26)**	0.003 (0.05)	1.05	0.09
	Speed/agility	3.23 (1.46–7.13)*	3.36 (1.54–7.32)*	0.08 (0.18)	1.31	2.4
	Flexibility	2.64 (1.48–4.72)*	2.94 (1.53–5.64)**	0.03 (0.15)	1.18	0.9
Sleep	General	1.11 (0.73–1.70)	1.10 (0.73–1.67)	0.02 (0.06)	1.14	0.6
	Muscular fitness	1.13 (0.70–1.80)	1.13 (0.73–1.75)	≈0	1.00	0.00
	Cardiorespiratory fitness	1.16 (0.72–1.85)	1.16 (0.75–1.80)	0.02 (0.12)	1.14	0.6
	Speed/agility	1.10 (0.72–1.68)	1.12 (0.74–1.71)	0.07 (0.12)	1.29	2.1
	Flexibility	1.18 (0.77–1.81)	1.20 (0.78–1.85)	0.07 (0.13)	1.29	2.1

OR, odds ratio; CI, confidence interval; SE, standard error; MOR, median odds ratio; ICC, intraclass correlation coefficient; ≈0, country-level variance was estimated as redundant indicating no between-country variability. Model 1, Adjusted for sex, age, breadwinner’s educational level; Model 2, adjusted for Model 1 + Countries. Significant values for *p* < 0.05.

*p < 0.05. **p < 0.001.

These associations remained significant in Model 2 after considering grouping by country. Not meeting ST recommendations was associated with lower odds of high cardiorespiratory fitness in the fully adjusted model (OR = 0.35; 95% CI: 0.12–0.96). Variability between countries was minimal (ICC < 5% for all domains), indicating that individual factors played a predominant role in explaining low physical fitness among adolescents.

Table 5 presents the associations between combined 24HMB and low self-reported physical fitness. Compared to meeting all three movement recommendations, meeting fewer components did not show a significant association with low physical fitness in any of the models.

**Table 5:** Associations between combined 24HMB and self-reported physical fitness in preschoolers, children, and adolescents.

24HMB	Outcome	Model 1 OR (95% CI)	Model 2 OR (95% CI)
Behavior (Ref: all three components)			
Two components	General	0.94 (0.24–3.63)	0.79 (0.21–3.01)
	Muscular fitness	0.78 (0.21–2.85)	0.64 (0.17–2.36)
	Cardiorespiratory fitness	0.47 (0.14–1.60)	0.39 (0.12–1.30)
	Speed/agility	1.00 (0.25–4.01)	0.96 (0.23–3.92)
	Flexibility	1.73 (0.40–7.42)	1.57 (0.37–6.59)
One component	General	1.93 (0.51–7.23)	1.68 (0.46–6.18)
	Muscular fitness	1.29 (0.36–4.58)	1.18 (0.33–4.21)
	Cardiorespiratory fitness	0.93 (0.28–3.05)	0.87 (0.28–2.77)
	Speed/agility	1.70 (0.44–6.62)	1.74 (0.44–6.91)
	Flexibility	2.59 (0.62–10.85)	2.55 (0.62–10.45)
None	General	1.98 (0.52–7.60)	1.73 (0.46–6.52)
	Muscular fitness	1.28 (0.35–4.70)	1.19 (0.33–4.37)
	Cardiorespiratory fitness	0.85 (0.25–2.88)	0.81 (0.25–2.64)
	Speed/agility	1.88 (0.47–7.50)	1.95 (0.48–7.92)
	Flexibility	2.60 (0.61–11.12)	2.58 (0.62–10.80)

24HMB	Outcome	Model 1 OR (95% CI)	Model 2 OR (95% CI)
Random effects (country)	Variance (SE)	MOR	ICC (%)
General	0.19 (0.17)	1.52	5.5
Muscular fitness	0.71 (0.47)	2.23	17.8
Cardiorespiratory fitness	0.03 (0.08)	1.18	0.9
Speed/agility	0.08 (0.10)	1.31	2.4
Flexibility	0.02 (0.03)	1.14	0.6

24HMB, 24-h movement behaviors; OR, odds ratio; CI, confidence interval; SE, standard error; MOR, median odds ratio; ICC, intraclass correlation coefficient; ≈0, country-level variance was estimated as redundant indicating no between-country variability. Model 1, Adjusted for sex, age, breadwinner’s educational level; Model 2, Adjusted for Model 1 + Countries. Significant values for *p* < 0.05.

*p < 0.05. **p < 0.001.

The associations between the sum of the 24HMB components and self-reported physical fitness varied according to age group (Supplementary Table S1). Among preschool children, no statistically significant associations were observed between the number of movement behavior recommendations met and any physical fitness domain in any of the models. In contrast, significant associations emerged among children, particularly after full adjustment (Model 2). Compared with those who met two components of movement behavior, children who met only one component showed higher odds of low general physical fitness (OR = 1.74; 95% CI: 1.06–2.85), cardiorespiratory fitness (OR = 1.88; 95% CI: 1.11–3.17), and speed/agility (OR = 1.73; 95% CI: 1.03–2.90). Additionally, children who did not meet any recommendation had a higher likelihood of low muscular fitness (OR = 2.36; 95% CI: 1.04–5.37). Among adolescents, associations were stronger and observed across multiple physical fitness domains. Adolescents who met only one recommendation demonstrated higher odds of low general physical fitness (OR = 1.68; 95% CI: 1.27–5.66), muscular fitness (OR = 2.48; 95% CI: 1.15–5.33), and cardiorespiratory fitness (OR = 2.54; 95% CI: 1.17–5.53). Those who did not meet any recommendations showed an even greater probability of unfavorable outcomes, including general physical fitness (OR = 2.81; 95% CI: 1.30–6.07), muscular fitness (OR = 2.45; 95% CI: 1.11–3.43), cardiorespiratory fitness (OR = 2.65; 95% CI: 1.19–5.93), speed/agility (OR = 2.30; 95% CI: 1.07–4.91), and flexibility (OR = 2.42; 95% CI: 1.15–5.11). Random-effects estimates indicated low between-country variability in the outcomes. Intraclass correlation coefficients were generally low, ranging from 0.0 to 11.8%, indicating that most of the variability in physical fitness was explained at the individual level rather than by differences between countries.

## 4 Discussion

The present study investigated the associations between the components of 24HMB (PA, ST, and sleep) and self-reported physical fitness in different age groups in an Ibero-American sample during the COVID-19 pandemic. Overall, PA emerged as the behavior most consistently associated with the outcomes of physical fitness, the associations becoming progressively stronger with increasing age. Although limited associations were observed among preschoolers, the failure to meet the PA recommendations was associated with poorer general physical fitness in this group and was consistently associated with multiple domains of low physical fitness among children and adolescents. In contrast, sleep and ST showed less consistent relationships with physical fitness outcomes. Furthermore, non-adherence to recommendations in combination was associated with less favorable physical fitness profiles, particularly in older age groups. The low intraclass correlation coefficients observed across the models further suggest that individual factors played a greater role in explaining variability in physical fitness than differences between countries.

A systematic review conducted by Zhang et al. (14) synthesized evidence on 24HMB during the COVID-19 pandemic and reported that fewer than 5% of children and adolescents met the combined movement behavior recommendations. Reductions in PA levels, increases in sedentary time, and changes in sleep patterns were largely attributed to social distancing policies and school closures (29–34). Evidence suggests that adherence to healthy behaviors tends to be lower during days without structured routines, such as school holidays, compared with structured school-day routines (35). Therefore, disruptions to daily routines during the pandemic likely contributed to the adoption of unhealthy behaviors observed across several countries. Increased access to electronic devices in children’s bedrooms may have further contributed to poorer sleep quality and duration as well as reduced daily PA levels (36). In this context, the prevalence of adherence to 24HMB guidelines observed across countries in the present study may reflect variations in public health restrictions and social distancing measures adopted. Overall, low adherence to ST recommendations was observed in all countries, particularly in Uruguay, whereas compliance with PA recommendations was more frequent in Uruguay and less frequent in Brazil. In contrast, sleep recommendations were more frequently met in Brazil and less frequently in Uruguay.

The current findings reinforce a growing body of evidence highlighting PA as the main behavioral determinant of physical fitness in youth (37, 38). Chen et al. (39) reported that adherence to the 24HMB guidelines was positively associated with multiple components of physical fitness, largely dependent on PA levels. Similarly, several authors argue that maintaining moderate-to-vigorous PA is the most relevant behavioral factor to preserve and improve cardiorespiratory and muscular fitness (38–40). Together, these findings suggest that PA plays a central role not only independently but also within the broader 24HMB framework. Our findings extend this evidence to the pandemic context, suggesting that PA may represent a priority target for interventions aimed at preserving physical fitness of young people during periods of social disruption (24, 41).

In contrast to PA, sleep duration and ST demonstrated weaker and less consistent associations with physical fitness outcomes. Although excessive screen exposure has frequently been associated with poorer physical fitness indicators in children and adolescents (42), Tanaka et al. (37) reported null associations between these factors. The authors also highlighted that the absence of association may be related to the low prevalence of children who meet the ST recommendations. This inconsistency across studies suggests that the relationship between these behaviors and physical fitness may depend on contextual and methodological factors. Sleep patterns during the pandemic also showed heterogeneous changes, some studies reporting increased sleep duration and others identifying sleep dysregulation. Evidence indicates that both insufficient and excessive sleep may be associated with poorer outcomes in physical fitness (43, 44), suggesting that sleep quality and regularity, rather than duration alone, may play a more relevant role.

Moraleda-Cibrián et al. (45) evaluated screen use and sleep patterns among Spanish adolescents during the first-wave lockdown and found that these two behaviors significantly affected other health-related habits, such as diet, body weight, sun exposure, and PA. Increased ST may contribute to reduced sleep time; however, other studies reported a longer sleep duration during the pandemic (46, 47). In their meta-analysis, Neville et al. (25) reported a significant reduction in PA among children and adolescents during the pandemic, much of which was associated with increased screen exposure. Together, these findings suggest that the 24HMB components may become imbalanced, raising concerns about the risks to healthy development.

When movement behavior components were analyzed jointly, the overall analysis showed no significant differences in physical fitness between those meeting all three guidelines and those meeting two, one, or none. However, when analyses were stratified by age group and meeting at least two guideline components was used as the reference category, meeting fewer 24HMB recommendations was associated with lower physical fitness levels, particularly among children and adolescents. Zhao et al. (48) demonstrated consistent evidence regarding the health implications of combined adherence to the three 24HMB guidelines in children and adolescents. Evidence indicates that meeting all three recommendations is associated with favorable health indicators, whereas meeting none is associated with unfavorable outcomes. Studies examining associations between overall adherence to 24HMB guidelines and physical fitness have shown that meeting all three recommendations is associated with higher general physical fitness, cardiorespiratory fitness, muscular fitness, speed, and agility (12, 37, 39, 49–51). According to Zhao et al. (48), studies examining specific combinations among the three guidelines primarily emphasize the importance of moderate-to-vigorous PA.

A systematic review conducted by Wilhite et al. (20), which evaluated associations between combinations of sedentary behavior, PA, and sleep with various health risk factors, identified studies in which children who engaged in more PA and less sedentary behavior demonstrated better results for muscular fitness. In the study by Tanaka et al. (37), children who met both recommendations for PA and sleep showed better results in muscular fitness and flexibility compared to those who met other combinations of movement behaviors. These findings reinforce the idea that different combinations of behaviors may influence specific components of physical fitness, although PA remains the most influential factor.

Understanding the combined associations of movement behaviors is extremely important for favorable physical and psychological health outcomes from childhood through adolescence. Overall people who are more physically active, less sedentary and who maintain adequate sleep duration patterns tend to have better health outcomes compared to their peers (20). These patterns highlight the importance of promoting balanced daily routines that integrate all movement behaviors. Therefore, importance cannot be attributed to only one behavior while ignoring the role of others. Furthermore, Ludwig-Walz et al. (24) emphasize that when evaluating physical fitness before, during, and after the pandemic, sex, age, and socioeconomic characteristics must be considered, as the results may vary according to the context.

A major strength of our study is the inclusion of preschoolers, children, and adolescents from different countries, providing a diverse sample and relevant evidence for public policies in different contexts. Additionally, this is one of the few studies that specifically evaluated 24HMB components both individually and in combination during a pandemic. However, some limitations should be acknowledged. Most of the studies conducted during the pandemic relied on self-reported assessments completed by parents or guardians. Therefore, potential social desirability and recall biases should be considered, as data were based on self-reports subject to responses influenced by social norms and possible inaccuracies. Differences in confinement restrictions imposed across countries may also have influenced both 24HMB and perceptions of physical fitness.

In conclusion, this study highlights physical activity as the central component of 24HMB associated with physical fitness, particularly in older age groups. While sleep and screen time showed less consistent relationships, the findings support the importance of considering movement behaviors in an integrated manner. Therefore, the results reinforce the need for integrated strategies that promote healthy behaviors throughout the day, prioritizing PA, especially during periods of disruption to daily routines, such as those observed during the pandemic.

## Data Availability

The data supporting the findings of this study are not publicly available due to ethical and privacy restrictions related to the inclusion of minors. Requests for access to the data may be considered by the corresponding author upon reasonable request and subject to applicable ethical and legal requirements.
