# Correction: Components of 24-hour movement behavior and self-reported physical fitness in Ibero-American preschoolers, children, and adolescents during the COVID-19 pandemic

**DOI:** 10.3389/fpubh.2026.1920429

**Published:** 2026-07-07

**Authors:** Frontiers Production Office

**Affiliations:** Frontiers Media SA, Lausanne, Switzerland

**Keywords:** physical condition, physical activity, sedentary behavior, sleep, youth

There was a mistake in Table 5 as published. The value for Flexibility and MOR was erroneously retained as 0.6 when it should be 1.14. The corrected Table 5 appears below.

**Table 5:** Associations between combined 24HMB and self-reported physical fitness in preschoolers, children, and adolescents.

24HMB	Outcome	Model 1 OR (95% CI)	Model 2 OR (95% CI)
Behavior (Ref: all three components)			
Two components	General	0.94 (0.24–3.63)	0.79 (0.21–3.01)
	Muscular fitness	0.78 (0.21–2.85)	0.64 (0.17–2.36)
	Cardiorespiratory fitness	0.47 (0.14–1.60)	0.39 (0.12–1.30)
	Speed/agility	1.00 (0.25–4.01)	0.96 (0.23–3.92)
	Flexibility	1.73 (0.40–7.42)	1.57 (0.37–6.59)
One component	General	1.93 (0.51–7.23)	1.68 (0.46–6.18)
	Muscular fitness	1.29 (0.36–4.58)	1.18 (0.33–4.21)
	Cardiorespiratory fitness	0.93 (0.28–3.05)	0.87 (0.28–2.77)
	Speed/agility	1.70 (0.44–6.62)	1.74 (0.44–6.91)
	Flexibility	2.59 (0.62–10.85)	2.55 (0.62–10.45)
None	General	1.98 (0.52–7.60)	1.73 (0.46–6.52)
	Muscular fitness	1.28 (0.35–4.70)	1.19 (0.33–4.37)
	Cardiorespiratory fitness	0.85 (0.25–2.88)	0.81 (0.25–2.64)
	Speed/agility	1.88 (0.47–7.50)	1.95 (0.48–7.92)
	Flexibility	2.60 (0.61–11.12)	2.58 (0.62–10.80)
**24HMB**	**Outcome**	**Model 1 OR (95% CI)**	**Model 2 OR (95% CI)**
**Random effects (country)**	**Variance (SE)**	**MOR**	**ICC (%)**
General	0.19 (0.17)	1.52	5.5
Muscular fitness	0.71 (0.47)	2.23	17.8
Cardiorespiratory fitness	0.03 (0.08)	1.18	0.9
Speed/agility	0.08 (0.10)	1.31	2.4
Flexibility	0.02 (0.03)	1.14	0.6

24HMB, 24-h movement behaviors; OR, odds ratio; CI, confidence interval; SE, standard error; MOR, median odds ratio; ICC, intraclass correlation coefficient; ≈0, country-level variance was estimated as redundant indicating no between-country variability. Model 1, Adjusted for sex, age, breadwinner's educational level; Model 2, Adjusted for Model 1 + Countries. Significant values for *p* < 0.05.

^*^p < 0.05. ^**^p < 0.001.

There was a mistake in the footnote of Table 5 as published. “Model 1, Adjusted by sex, age, breadwinner's educational level; Model 2, Adjusted by Model 1 + Countries.” should read “Model 1, Adjusted for sex, age, breadwinner's educational level; Model 2, Adjusted for Model 1 + Countries.” The corrected footnote of Table 5 appears below.

The original version of this article has been updated.

