# Supplementary material for: Components of 24-hour movement behavior and self-reported physical fitness in Ibero-American preschoolers, children, and adolescents during the COVID-19 pandemic
Source: Front Public Health. 2026 Jun 11;14:1837281. doi: 10.3389/fpubh.2026.1837281 (PMC13294265; doi:10.3389/fpubh.2026.1837281)
Supplement: Supplementary file 1 [file Table_1.DOCX]

Supplementary Material

**Supplementary table.** Associations between combined 24HMB and self-reported physical fitness in preschoolers, children, and adolescents.

|  | **Outcome** | **Model 1**  **OR (95% CI)** | | | **Model 2**  **OR (95% CI)** | | |
| --- | --- | --- | --- | --- | --- | --- | --- |
| **Preschoolers** |  |  |  |  |  |  |  |
| *Behavior (Ref: meets two components)* | |  | | |  | | |
| **One component** | General | 2.09 (0.57 – 7.60) | | | 1.95 (0.64 – 5.93) | | |
|  | Muscular fitness | 1.51 (0.58 – 3.92) | | | 1.58 (0.47 – 5.27) | | |
|  | Cardiorespiratory fitness | 2.07 (0.67 – 6.24) | | | 2.25 (0.77 – 6.63) | | |
|  | Speed/agility | 2.02 (0.64 – 6.32) | | | 2.40 (0.65 – 8.82) | | |
|  | Flexibility | 2.05 (0.50 – 8.88) | | | 2.21 (0.58 – 8.37) | | |
| **None** | General | 2.32 (0.45 – 11.79) | | | 2.47 (0.60 – 10.06) | | |
|  | Muscular fitness | 0.45 (0.08 – 2.37) | | | 0.40 (0.05 – 3.36) | | |
|  | Cardiorespiratory fitness | 1.22 (0.24 – 6.08) | | | 1.29 (0.27 – 6.08) | | |
|  | Speed/agility | 1.46 (0.30 – 7.17) | | | 1.54 (0.26 – 9.26) | | |
|  | Flexibility | 0.99 (0.12 – 7.87) | | | 1.06 (0.15 – 7.32) | | |
| *Random effects (country)* |  | Variance (SE) | MOR | | | ICC (%) | |
|  | General | 0.29 (0.43) | 1.67 | | | 8.1 | |
|  | Muscular fitness | 0.44 (0.58) | 1.88 | | | 11.8 | |
|  | Cardiorespiratory fitness | 0.004 (0.65) | 1.06 | | | 0.12 | |
|  | Speed/agility | 0.13 (1.23) | 1.41 | | | 3.8 | |
|  | Flexibility | ≈0 | 1.00 | | | 0.00 | |
| **Children**  *Behavior (Ref: meets two components)* | **Outcome** | **Model 1**  **OR (95% CI)** | | | **Model 2**  **OR (95% CI)** | | |
| **One component** | General | 1.68 (1.05 – 2.69)* | | | 1.74 (1.06 – 2.85)* | | |
|  | Muscular fitness | 1.45 (0.89 – 2.34) | | | 1.66 (0.98 – 2.81) | | |
|  | Cardiorespiratory fitness | 1.62 (0.98 – 2.65) | | | 1.88 (1.11 – 3.17)* | | |
|  | Speed/agility | 1.56 (0.94 – 2.57) | | | 1.73 (1.03 – 2.90)* | | |
|  | Flexibility | 1.40 (0.90 – 2.18) | | | 1.55 (0.98 – 2.46) | | |
| **None** | General | 1.53 (0.72 – 3.24) | | | 1.60 (0.72 – 3.52) | | |
|  | Muscular fitness | 2.22 (1.04 – 4.72)* | | | 2.36 (1.04 – 5.37)* | | |
|  | Cardiorespiratory fitness | 1.50 (0.68 – 3.33) | | | 1.52 (0.66 – 3.47) | | |
|  | Speed/agility | 1.98 (0.90 – 4.34) | | | 1.97 (0.88 – 4.43) | | |
|  | Flexibility | 1.13 (0.54 – 2.37) | | | 1.11 (0.52 – 2.39) | | |
| *Random effects (country)* |  | Variance (SE) | MOR | | | | ICC (%) |
|  | General | 0.02 (0.09) | 1.14 | | | | 0.6 |
|  | Muscular fitness | 0.11 (0.15) | 1.37 | | | | 3.2 |
|  | Cardiorespiratory fitness | 0.14 (0.17) | 1.43 | | | | 4.1 |
|  | Speed/agility | 0.16 (0.16) | 1.47 | | | | 4.6 |
|  | Flexibility | 0.03 (0.07) | 1.18 | | | | 0.9 |
| **Adolescents**  *Behavior (Ref: meets two components)* | **Outcome** | **Model 1**  **OR (95% CI)** | **Model 2**  **OR (95% CI)** | | | | |
| **One component** | General | 2.68 (1.28 – 5.61)* | | | 2.68 (1.27 – 5.66)* | | |
|  | Muscular fitness | 2.42 (1.09 – 5.38)* | | | 2.48 (1.15 – 5.33)* | | |
|  | Cardiorespiratory fitness | 2.39 (1.04 – 5.49)* | | | 2.54 (1.17 – 5.53)* | | |
|  | Speed/agility | 1.83 (0.89 – 3.80) | | | 1.78 (0.85 – 3.71) | | |
|  | Flexibility | 1.66 (0.81 – 3.39) | | | 1.69 (0.83 – 3.44) | | |
| **None** | General | 2.88 (1.35 – 6.19)* | | | 2.81 (1.30 – 6.07)* | | |
|  | Muscular fitness | 2.38 (1.04 – 5.45)* | | | 2.45 (1.11 – 5.43)* | | |
|  | Cardiorespiratory fitness | 2.46 (1.04 – 5.80)* | | | 2.65 (1.19 – 5.93)* | | |
|  | Speed/agility | 2.24 (1.06 – 4.76)* | | | 2.30 (1.07 – 4.91)* | | |
|  | Flexibility | 2.27 (1.07 – 4.79)* | | | 2.42 (1.15 – 5.11)* | | |
| *Random effects (country)* |  | Variance (SE) | | MOR | | | ICC (%) |
|  | General | 0.01 (0.06) | | 1.10 | | | 0.3 |
|  | Muscular fitness | ≈0 | | 1.00 | | | 0.00 |
|  | Cardiorespiratory fitness | 0.005 (0.05) | | 1.07 | | | 0.15 |
|  | Speed/agility | 0.11 (0.75) | | 1.37 | | | 3.2 |
|  | Flexibility | 0.08 (0.13) | | 1.31 | | | 2.4 |

OR= odds ratio. CI= confidence interval. SE = standard error. MOR = median odds ratio. ICC = intraclass correlation coefficient. ≈0 = country-level variance was estimated as redundant indicating no between-country variability. Model 1: Adjusted by sex, age, breadwinner’s educational level; Model 2: Adjusted by Model 1 + Countries. Significant values for p<0.05.

*p<0.05. **p<0.001.
